# CA-125–indicated asymptomatic relapse confers survival benefit to ovarian cancer patients who underwent secondary cytoreduction surgery

**DOI:** 10.1186/1757-2215-6-14

**Published:** 2013-02-13

**Authors:** Fang Wang, Yanfen Ye, Xia Xu, Xuehui Zhou, Jinhua Wang, Xiaoxiang Chen

**Affiliations:** 1 Research Institute of Obstetrics and Gynecology, The third Affiliated Hospital of Guangzhou Medical University, Guangzhou, Guangdong, 510150, PR China; 2 Department of Pathology, The University of Texas MD Anderson Cancer Center, Houston, TX, 77030, USA; 3 Cancer Research Institute, Southern Medical University, Guangzhou, 510515, PR China; 4 Department of Chemotherapy, Jiangsu Cancer Hospital, Nanjing, Jiangsu, 210009, PR China; 5 Department of Gynecologic Oncology, Jiangsu Cancer Hospital, Nanjing, Jiangsu, 210009, PR China; 6 State Key Laboratory of Bioelectronics, South University, Nanjing, 210096, PR China

**Keywords:** Epithelial ovarian cancer, CA-125, Tumor marker, Clinical relapse, Cytoreductive surgery

## Abstract

**Background:**

There is no consensus regarding the management of ovarian cancer patients, who have shown complete clinical response (CCR) to primary therapy and have rising cancer antigen CA-125 levels but have no symptoms of recurrent disease. The present study aims to determine whether follow-up CA-125 levels can be used to identify the need for imaging studies and secondary cytoreductive surgery (CRS).

**Methods:**

We identified 410 ovarian cancer patients treated at The University of Texas MD Anderson Cancer Center between 1984 and 2011. These patients had shown CCR to primary therapy. Follow-up was conducted based on the surveillance protocol of the MD Anderson Cancer Center. We used the Cox proportional hazards model and log-rank test to assess the associations between the follow-up CA-125 levels and secondary CRS and survival duration.

**Results:**

The CA-125 level of 1.68 × nadir was defined as the indicator of recurrent disease (p < 0.001). The specificity and sensitivity of this criterion were 82.9% and 85.6%, respectively, and the median lead-time of the CA-125 biochemical progression prior to clinically-defined relapse was 31 days (ranging from 1 to 391 days). The median number of the negative imaging studies for the clinical relapse findings in patients with a CA-125 level of < 1.68 × nadir was 3 (ranging from 0 to 24 times). The increase of CA-125 level at relapse was an independent predictor of overall and progression free survival in patients who had shown CCR to primary therapy (p = 0.04 and 0.02 respectively). The overall and progression free survival durations in patients with a CA-125 level ≤ 1.68 × nadir at relapse (69.4 and 13.8 months) were longer than those with a CA-125 level > 1.68 × nadir at relapse (55.7 and 10.4 months; p = 0.04 and 0.01, respectively). The overall and progression free survival duration of patients with asymptomatic relapse and underwent a secondary CRS was longer than that of patients with symptomatic relapse (p = 0.02 and 0.04 respectively).

**Conclusions:**

The increase of serum CA-125 levels is an early warning of clinical relapse in ovarian cancer. Using CA-125 levels in guiding the treatment of patients with asymptomatic recurrent ovarian cancer, who have shown CCR to primary therapy, can facilitate optimal secondary CRS and extend the survival duration of the patients.

## Background

Epithelial ovarian cancer (EOC) is the most lethal gynecologic malignancy in the United States [1]. Although primary therapy results in a complete clinical response (CCR) in more than 50% of EOC patients, achieving complete cure is rare. In fact, 3 out of 4 EOC patients develop recurrent disease within 2 years and ultimately die from the disease within 5 years [2]. The early detection and treatment of recurrent EOC improves the survival outcome of patients; however, the absence of effective follow-up enabling early detection and treatment remains a clinical challenge. Moreover, there is no agreement regarding the characteristics that indicate recurrence, the surveillance interval, and the necessary means of follow-up [3-6]. Unlike many other recurrent solid tumors, which have macroscopic masses, a significant proportion of recurrent EOC presents as immeasurable micro-nodular lesions and ascites that are widespread and diffused; therefore, quantifying recurrence in time is as difficult as seeking effective therapy in EOC patients.

The CA-125 antigen is a serum marker first identified by Bast et al. and developed through the immunization of mice by a monoclonal antibody (OC125), using an ovarian cancer cell line in 1981. Since then, CA-125 has been validated as an effective marker for monitoring EOC [7]. Rustin et al. posited that the criterion for tumor progression in the detection of recurrent EOC is a CA-125 level of 2 × the upper limit of normal (ULN), which has a sensitivity and specificity of 88% and 98%, respectively. This CA-125 threshold is accepted by the Gynecologic Cancer InterGroup [8-12]. Only one randomized trial has been conducted to investigate the value of routinely measuring follow-up CA-125 levels in EOC patients. In the MRC OV05/EORTC 55955 trial, Rustin et al. suggested that reintroducing chemotherapy in patients who have shown signs of biochemical recurrence of the disease does not improve survival duration, and that patients should not undergo regular CA-125 measurements if they have no symptoms suggesting relapse [13]. The value of routine CA-125 follow-up was thus denied. However, this trial did not rule out the possibility of using CA-125 levels to guide the early detection of clinically recurrent EOC, which can benefit from the earlier administration of secondary cytoreductive surgery (CRS) [14].

Secondary CRS is another major treatment for recurrent EOC. The DESKTOP I and II trials identified three independent factors for indicating complete resection, namely, good performance status, complete resection at primary surgery, and the absence of ascites. Complete resection when all three factors are present reached 79% in the DESKTOP I trail and 76% in the DESKTOP II trail [15,16]. The prospective DESKTOP III study on this issue is ongoing. Identifying the appropriate patients for the potentially morbidity-inducing procedure remains a clinical challenge. Hence, the possibility of utilizing rising CA-125 levels to optimize the secondary CRS in asymptomatic EOC patients with recurrent disease remains uncertain.

Prior to the appearance of a measurable disease on imaging studies, CA-125 levels rise a median of 4 months in approximately 70% of EOC patients who develop a recurrent disease [11,17]. The criterion used to define EOC recurrence has remained relatively stable over the past 20 years; in comparison, the density resolution technology in medical imaging has improved significantly [18-20]. The widespread use of higher-resolution Computed Tomography (CT), Magnetic Resonance Imaging (MRI), and Positron Emission Tomography-Computed Tomography (PET-CT) has resulted in the more timely and accurate detection of smaller lesions. The use of sensitivity, specificity, and lead-times attained using a CA-125 level of 2 × ULN as part of the criterion in defining recurrent disease in EOC patients was formulated using radiological technology nearly two decades ago. However, the relevance of such criterion remains uncertain.

In the present study, we retrospectively evaluated EOC patients who achieved CCR through primary therapy without undergoing consolidation and maintenance therapies. The CA-125 levels recorded after completing primary therapy were used as indicators of clinical relapse. The overall survival (OS) and progression free survival (PFS) of patients receiving secondary CRS, as indicated by the CA-125 levels or the relapse-related symptoms, were analyzed. Our data revealed the value of CA-125 follow-up in patients with the opportunity for further surgery.

## Methods

### Study population

This retrospective study was approved by the Institutional Review Board of MD Anderson. We identified 410 EOC patients who achieved CCR following primary treatment at MD Anderson from clinical stations between January 1, 1984 and February 14, 2011. Those who did not receive the standard primary treatment and achieved CCR were excluded. When the patients completed primary therapy, follow-up was conducted based on the routine follow-up protocol for EOC of MD Anderson, which included follow-up visits every 3 months for the first 2 years, every 4 months in year 3, and every 6 months in years 4 and 5. The patients underwent medical history investigation, physical examination, and CA-125 tests at each follow-up visit. Imaging studies (i.e., ultrasonography, MRI, or area-specific CT or x-ray) were performed in all patients at the first follow-up visit; thereafter, imaging studies were performed only in patients whose clinical examinations suggested recurrent cancer. The Response Evaluation Criteria in Solid Tumors (RECIST) and the World Health Organization (WHO) criteria were used to assess tumor therapy response and clinical relapse [21-23].

We evaluated the clinicopathological data of all patients, including the histological type and grade of the tumor [24,25], stage of the disease as defined by the International Federation of Gynecology and Obstetrics [26], type of primary treatment received, volume of ascites, time of recurrent disease diagnosis, management of recurrent disease, and date of death or last follow-up. The OS duration was defined as the time beginning from the diagnosis of EOC to death or last follow-up. PFS was the length of time after treatment during which patients being treated does not get worse. Pathological diagnoses of recurrent EOC were reviewed at the time of diagnosis by two MD Anderson pathologists, namely, J. Liu and J. Zhang.

### Definition of clinical response and CA-125 analysis

In the current study, CCR was achieved when the following criteria were met: the absence of tumor-associated clinical symptoms, signs of residual tumor on the physical examination and EOC-negative imaging study results, and a serum CA-125 concentration below the ULN. Clinical relapse was identified with the occurrence of any new measurable lesion, as revealed through imaging studies or clinical examination [22]. Biochemical relapse was assessed using CA-125 levels and then identified based on the receiver operating characteristic (ROC) curve.

Next, we reviewed the serum CA-125 concentrations recorded from the end of primary therapy to death or last follow-up. The serum CA-125 concentrations were determined using a commercially available Roche immunoassay. In order to determine the nadir CA-125 levels of individual patients, we included the CA-125 levels recorded in the 2 weeks following the first follow-up evaluation after the completion of primary therapy. The CA-125 recorded at relapse included those recorded in the 2 weeks following the confirmation of imaging-defined recurrent EOC. The apex of the follow-up CA-125 level was defined as the highest CA-125 level whose measurement can be confirmed (i.e., repeated with < 25% variation in the result) within 3 months in EOC patients without recurrent disease. Tumor-associated symptomatic relapse was defined as the recurrence of the disease after returning to MD Anderson for unscheduled follow-up visits were considered to have tumor-associated symptoms. Secondary CRS as a selective procedure was performed in 84 Platinum-sensitive EOC patients (generally referring to the progression of the free interval of at least 6 months from the completion of primary therapy) with good performance status and intended purpose of tumor reduction. Optimal cytoreduction was defined as the threshold of ≤ 1 cm of the residual tumor, and suboptimal debulking was determined as having more than 1 cm of nodules left.

### Statistical analysis

We used the Cox proportional hazards model to assess the relationship between the CA-125 level as a continuous variable and the OS and PFS. Step-wise regression was conducted to build the multivariate models. The log-rank test was used to assess this relationship as a dichotomous variable around the median value. We also calculated the ROC curve, which was placed over the data points to form a smoothed curve using a regression model. Next, we performed the Z-test to determine whether the sensitivity and/or specificity of one modality were significantly different from that of the others. The p values < 0.05 were considered statistically significant. All analyses were conducted using the SPSS statistical software program (version 18.0; SSPS Inc, Chicago, IL).

## Results

### Patient characteristics

**Figure 1:**
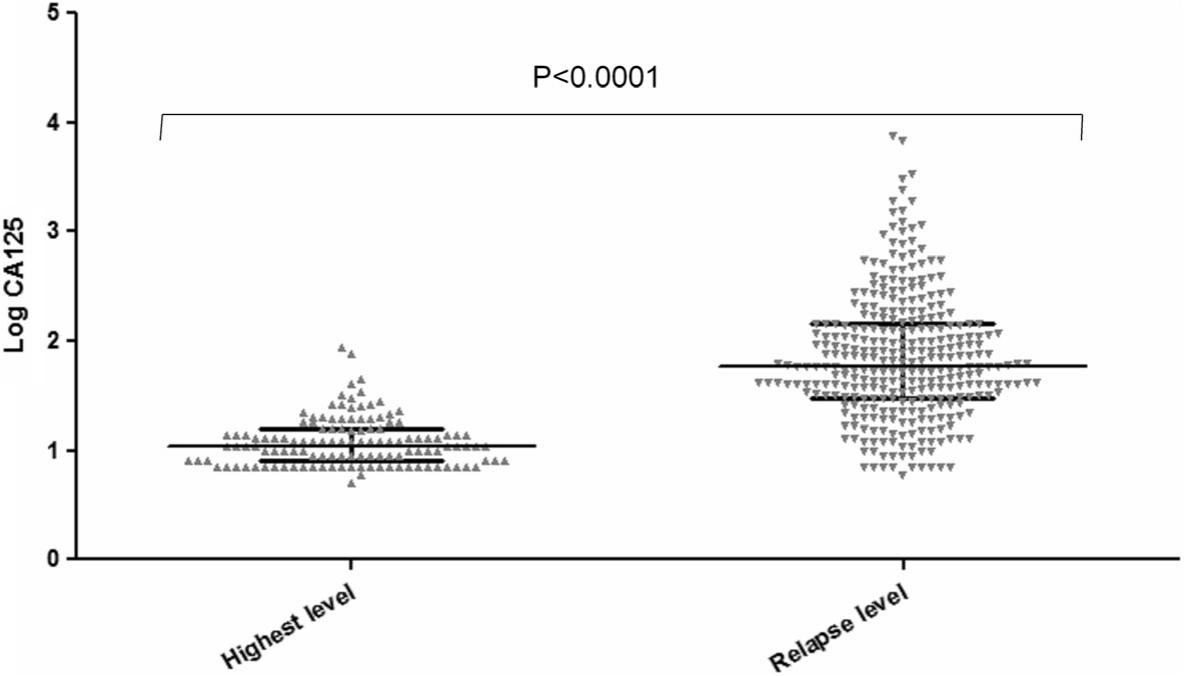
Serum CA-125 concentrations in patients who developed recurrent EOC (median, 53 U/ml; range, 6 U/ml to 3372 U/ml) were significantly higher than the highest CA-125 level recorded during follow-up in patients who did not develop recurrent disease (median, 11 U/ml; range, 5 U/ml to 87 U/ml; p < 0.001).

The clinicopathological characteristics of the patients are given in Table 1. Of the 410 EOC patients included in the present study, 361 (88.0%) and 49 (12.0%) patients had high-grade and low-grade primary tumors, respectively. Most patients (317; 77.3%) had serous carcinoma. The patients who were alive at the time of our analysis had a median follow-up time of 38 months (interquartile range, 20.4 months to 73.2 months). The median OS duration of all the patients was 72.1 months (95% confidence interval [CI], 63.4 months to 80.8 months). The median CA-125 level at relapse (53 U/ml; range, 6 U/ml to 3372 U/ml) was significantly higher than that of the median pre-relapse highest CA-125 level (11 U/ml; range, 5 U/ml to 87 U/ml; p < 0.001; Figure 1). The patients who were symptomatic at recurrence reported experiencing pain (8 patients), gastrointestinal dysfunction (26 patients), and/or mass effect (13 patients) and underwent secondary CRS.

**Table 1:** Patient characteristics of the present study population

**Characteristic**	**No. of patients (%)**
Median age, years (range)	58.6 (23.5–89.2)
Median CA-125 level at diagnosis, U/ml (range)	490.5 (7–33439)
Median nadir CA-125 level, U/ml (range)	10 (4–35)
Ethnicity	
White	316 (77.1)
Black	25 (6.1)
Hispanic	56 (13.7)
Other*	13 (3.2)
Histology	
Serous	317 (77.3)
Endometrioid	42 (10.2)
Clear cell	18 (4.4)
Mucinous	9 (2.2)
Transitional	6 (1.5)
Undifferentiated	9 (2.2)
MMMT	9 (2.2)
Grade	
High	361 (88.0)
Low	49 (12.0)
FIGO stage	
I	42 (10.2)
II	31 (7.6)
III	284 (69.3)
IV	53 (12.9)
Residual disease after primary surgery	
Optimal (**≤** 1 cm)	322
Suboptimal (> 1 cm)	65
Unknown	23
NACT	
Yes	46 (11.2)
No	354 (86.3)
Unknown	10 (2.4)

Note: All data are no. of patients (%) unless noted otherwise.

Abbreviations: *MMMT*, malignant mixed müllerian tumor; *FIGO*, International Federation of Gynecology and Obstetrics; *NACT*, Neoadjuvant chemotherapy.

*5 Chinese, 4 Middle Eastern, 1 Japanese, 1 Korean, and 2 Indian patients.

### Prediction of clinical relapse using follow-up CA-125 level

**Figure 2:**
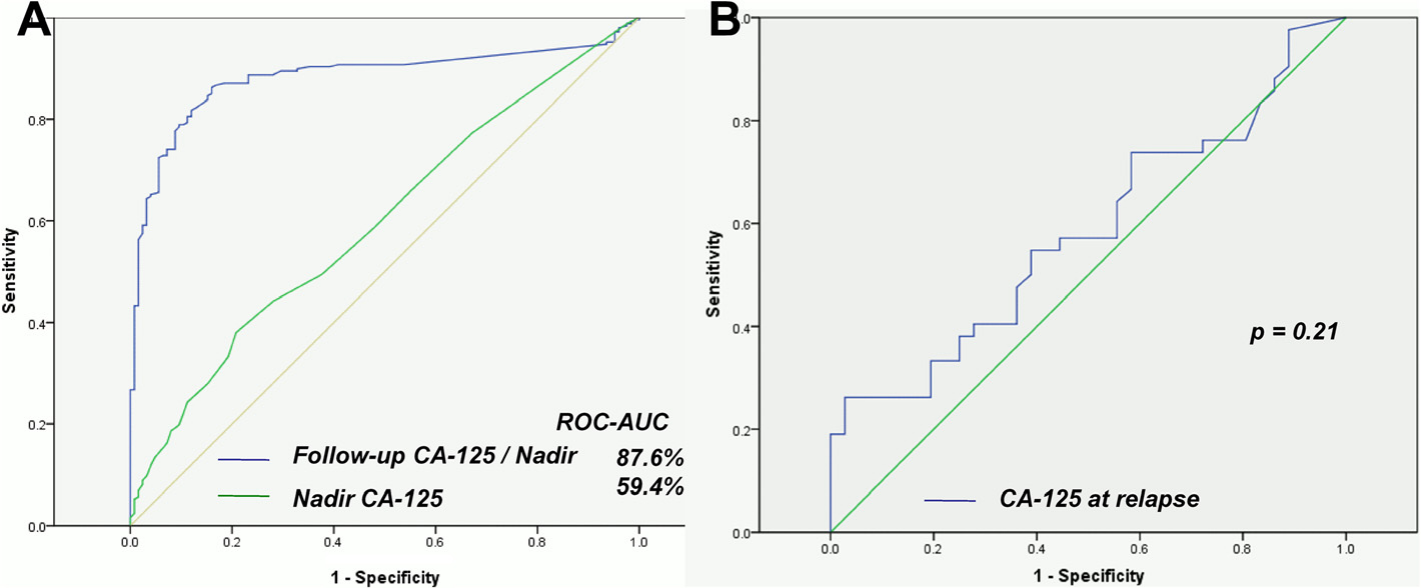
ROC curve for tumor relapse (A) and optimal secondary CRS (B) by CA-125 level.

To predict clinical relapse, we used the ROC curve to analyze the highest CA-125 levels during the follow-up sessions in patients who did not have recurrence and had relapse (p < 0.001). The sensitivity and the specificity for predicting clinical relapse were 82.9% and 85.6%, respectively, which were identified using a CA-125 level of 1.68 × nadir as the cut-off point (Figure 2A).

Of the 193 patients who had clinical recurrence, 111 (57.5%) had an increased CA-125 level to 1.68 × nadir before clinical recurrence; the median lead-time in these patients was 31 days (range, 1 to 391 days). Of the 106 patients who had shown biochemistry relapse, 42 (39.6%) had an increased CA-125 level to 2 × ULN before clinical recurrence; the median lead-time in these patients was 16 days (range, 1 to 250 days). When the CA-125 level was at 2 × the ULN, the sensitivity and specificity for predicting clinical relapse were 36.9% and 98.4% respectively. Finally, the median interval was 22 days (range 2 to 68 days, Table 2) when the CA-125 level increased from 1.68 × nadir to 2 × the ULN.

**Table 2:** Relapse prediction using the rising values of serum CA-125 levels from nadir to ≥ 1.68 × nadir or to ≥ 70 U/ml

**Variable**	**1.68 × Nadir**			**70 U/ml**
	**CT**	**MRI**	**PET–CT**	
True positive, no. of patients	170	6	14	90
False positive, no. of patients	11	0	2	2
True negative, no. of patients	115	6	2	124
False negative, no. of patients	74	3	8	154
Sensitivity, %	69.7	66.6	63.6	36.9
Specificity, %	88.5	100	50.0	98.4
Positive predictive value, %	93.9	100	87.5	97.8
Negative predictive value, %	60.8	66.6	20.0	44.6
Median lead-time, days (range)	31 (1–391)			16 (1–250)
Median lead-time from 2 × nadir to 70 U/ml, days (range)	22 (0–46)			

Abbreviations: *CT*, computed tomography; *MRI*, magnetic resonance imaging; *PET*, positron emission tomography.

On average, the patients underwent imaging checks (CT, MRI, and/or PET-CT) thrice (range, 0 to 24 imaging studies) before clinical relapse. The median CA-125 measurement time was 16 (range, 0 to 52 measurements) at the same periods.

### Determining the association of CA-125 level with tumor size and optimal secondary CRS

The median longest tumor diameter at relapse was 2.1 cm (range, < 1 cm to 14 cm), and the median area of the tumor at relapse was 5.3 cm^2^ (range, < 1 cm^2^ to 144 cm^2^); these were obtained through imaging assays. There were no correlations between CA-125 level on the one hand and the longest diameter of the tumor based on RECIST (p = 0.21) and the tumor area based on the WHO criteria (p = 0.35) on the other hand. Likewise, pre-surgery CA-125 level (p = 0.21) was not associated with optimal secondary CRS (Figure 2B).

### Identifying CA-125 level and secondary CRS as independent survival indicators

Cox proportional hazards model revealed that the increase of CA-125 level at relapse were independent OS and PFS predictors of recurrent EOC (p = 0.04 and 0.02 respectively; Table 3).

**Table 3:** Hazard ratios of survival through the clinicopathological characteristics by multivariate analysis in recurrent ovarian cancer patients

**Characteristic**	**Overall survival**		**Progression free survival**	
	**HR**	**95% CI**	**HR**	**95% CI**
**FIGO stage**				
I	1.000	Reference	1.00	Reference
II	1.04	0.44–2.44	1.08	0.68-1.73
III	1.62	0.67–4.88	1.21	0.27-5.40
IV	2.27	1.83–6.69	1.72	1.66-4.46
Grade	1.93	0.84–4.36	2.49	1.05-2.94
Primary CRS	1.06	0.58–2.86	1.65	1.08–2.86
Secondary CRS	1.41	1.01–2.10	1.77	1.14-2.74
Ascites	1.79	1.05–3.04	1.83	1.03-3.27
Relapse/Nadir CA-125 level	1.00	1.00–1.01	1.00	1.00-1.01

Abbreviations: *HR*, hazard ratio; *CI*, confidence interval; *FIGO*, International Federation of Gynecology and Obstetrics; *CRS*, cytoreductive surgery.

**Figure 3:**
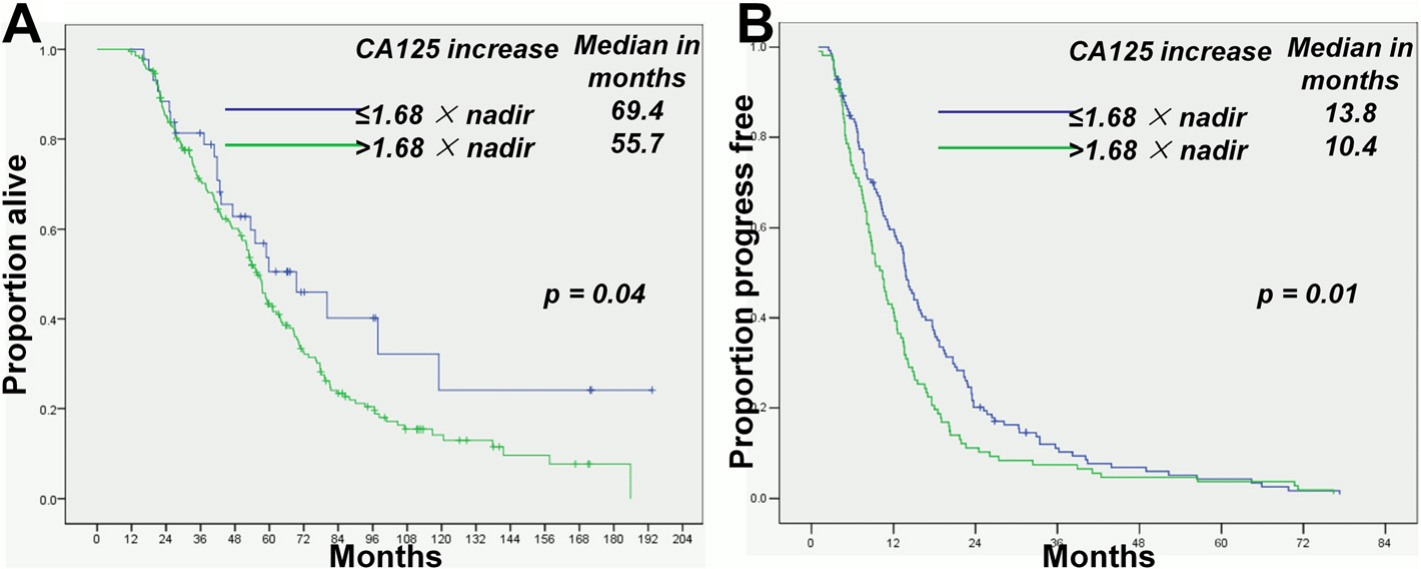
OS (A) and PFS (B) durations were shorter in patients with a CA-125 level of > 1.68 × nadir at relapse than those in patients with a CA-125 level of ≤ 1.68 × nadir at relapse.

**Figure 4:**
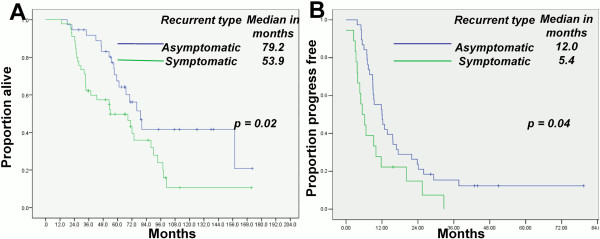
Symptomatic patients who underwent secondary CRS had shorter OS (A) and PFS (B) durations than asymptomatic patients.

The median OS and PFS durations of patients with a serum CA-125 concentration of ≤ 1.68 × nadir at relapse (69.4 and 13.8 months, respectively) were significantly longer than those of patients with a serum CA-125 level of > 1.68 × nadir at relapse (55.7 and 10.4 months; p = 0.04 and 0.01 respectively; Figure 3A and B). Multivariate analyses revealed that secondary CRS was an independent predictor of OS and PFS (p = 0.02 and p = 0.01 respectively; Table 3). The OS durations of patients who underwent secondary and optimal CRS were longer than those of patients who did not undergo or had suboptimal surgery (p = 0.01 and p = 0.04 respectively; Additional file 1: Figure S1A and B), Likewise, the PFS durations (p = 0.01 and p = 0.02 respectively; Additional file 2: Figure S2A and B). Asymptomatic patients in whom the recurrent disease was defined through a CA-125 level had a higher optimal secondary CRS rate (92.2% vs 43.5%) and longer OS duration than the symptomatic patients (p = 0.02; Figure 4A). The PFS duration of the patients who underwent optimal secondary CRS for the asymptomatic recurrent patients was significantly longer than that of the symptomatic recurrent cases (p = 0.04; Figure 4B).

## Discussion

The mean 5-year survival rate following the diagnosis of recurrent EOC is less than 10%. The management of recurrent diseases is a key aspect in the overall therapeutic management of patients with EOC. In addition to determining the most appropriate treatment for the primary disease, defining the triggers that signify the need for initiating or changing therapy at relapse is important in ensuring effective management [27,28]. The findings of the present study were focused on the use of CA-125 levels to guide the treatment of asymptomatic recurrent EOC patients who had shown CCR to primary therapy. We found that CA-125 follow-up can facilitate optimal secondary CRS and extend the OS durations of patients.

Many previous studies on this topic ignored secondary CRS, which is one of the major treatment choices apart from chemotherapy in selected cases. A previous study revealed that tumor burden was an independent factor associated with therapy response and survival [29]. Regular follow-up once a patient who has undergone standard primary treatment has achieved CCR is warranted, because a patient who develops recurrent EOC can benefit from being treated without delay when their tumors are comparatively small.

The MRC OV05/EORTC 55955 trial showed contrasting results with those that reported that the earlier initiation of second-line chemotherapy for recurrent EOC patients can improve their outcomes as testified by some other studies [30,31]. Secondary CRS has also been considered to be appropriate for patients with a low-volume disease or isolated lesions. Some retrospective studies have suggested that select patients who undergo optimal secondary CRS have better PFS and OS duration than those who do not undergo therapy [14,32,33].

Several factors influence whether recurrent lesions can be optimally resected, including the number and size of the tumors, the presence of ascites and/or carcinomatosis, and progression-free interval. CA-125 levels can help to recruit populations for secondary debulking. Fleming et al. showed that a shorter interval between the CA-125 elevation and the secondary CRS correlates with a greater incidence of optimal resection, thus leading to a longer median OS duration (47 months) than that of longer interval cases (23 months) [32]. They also found that the secondary CRS was delayed every week in the 16.4 weeks following the first elevation in CA-125 level; furthermore, the likelihood that the resection would be complete decreased by 3%. Frederick et al. proposed that a preoperative CA-125 level of < 250 U/ml can predict surgical success leading to prolonged survival [34]. In the present study, we found that the CA-125 level-guided asymptomatic recurrent EOCs have higher secondary CRS rates and longer OS and PFS durations than those in symptoms that determined relapse.

Recurrent EOC, unlike other solid tumors, tends to present without accompanying symptoms and forms multiple small implantations, particularly in the small intestine and mesentery, which cannot be readily detected using conventional imaging examinations [35,36]. The RECIST and WHO criteria for recurrent EOC focus on detectable changes in tumor size rather than the metastatic characteristics of the disease. However, imaging study results may not accurately reflect disease progression, because the microlesions are difficult to detect using such studies. The CA-125 level is an alternative indicator of tumor relapse, and is a surrogate of imaging study-defined relapse. The GCIG criterion is initially effective in identifying asymptomatic patients with recurrent EOC with sufficient sensitivity and an extremely low false positive rate (specificity > 97%) while avoiding the unnecessary treatment of patients who do not have recurrent disease. However, the sensitivity of these biochemical relapse criteria has since decreased to 36.9%. The resolutions attainable through imaging systems have improved dramatically over the past two decades. Imaging-based relapse criteria are confounded by the use of low- or high-density resolution imaging studies to detect smaller lesions [37]. Other imaging technologies, such as PET-CT, dynamic contrast-enhanced MRI, diffusion-weighted MRI and perfusion CT, have further elevated the resolution efficiency, accurately detecting the presence of microlesions. The combination of image-guided biopsy and a lower CA-125 level can be used to detect microlesions. In the present study, the sensitivity attained using a CA-125 level of 1.68 × nadir was relatively high (85.6%).

Ovarian cancer patients who have CCR to primary therapy are not a homogenous group. The nadir CA-125 level is a factor in determining patient prognosis. A slight increase in the CA-125 levels within the normal range at the end of primary treatment is inversely associated with that of PFS and OS. In the present study, the time during which the CA-125 levels increased from different individual nadir value to 2 × ULN was significantly different. The comparison between the threshold CA-125 level defined by the GCIG and the absolute CA-125 values without taking individual nadir CA-125 levels is considered in our criterion.

The continuous increase of CA-125 levels implies a continuous dominant course of tumor progression. Determining a CA-125 level at which the recurrent disease would be distinguishable using imaging studies is important. In the present study, this CA-125 value was determined on the basis of its use in diagnosing recurrent EOC. In contrast to the data presented by previous studies, the potential cut-off CA-125 values have been tested using their diagnostic performance and areas under the ROC curve.

An increase in CA-125 levels in an asymptomatic patient presents a dilemma. Some gynecologic oncologists recommend a wait-and-see approach, but the duration of the waiting period and how the patients should be monitored are unclear. Asymptomatic patients waiting for symptoms can experience significant anxiety and report unspecific symptoms to their physicians, thereby resulting in unnecessary imaging examinations. Regular CA-125 testing might also give rise to the psychosocial impairments of the patients based on the possible rise of CA-125 levels during their daily lives. A clear clinical interventional point of the CA-125 level can thus help patients overcome CA-125 addiction to a certain extent only if they prefer to not know the specifics. Other gynecologic oncologists argued that time and treatment choices are lost from asymptomatic to symptomatic. A CA-125 level of 1.68 × nadir in asymptomatic patients undergoing a wait-and-see approach can be used as a threshold at which specific imaging examinations become warranted to avoid missing the opportunity to perform secondary CRS. However, our findings did not reveal a relationship between the CA-125 levels at disease relapse and the imaging study-determined longest diameters of tumors or tumor areas.

Given the selection bias inherent to non-blinded studies, attempts to translate these favorable outcomes to all EOC patients with recurrent disease should be approached with caution. We recommend using a CA-125 level of 1.68 × nadir to indicate the need to initiate imaging studies in detecting recurrent disease in EOC patients who had CCR to primary therapy. Using this criterion would result in fewer instances of specious relapse and unnecessary imaging examinations. The ongoing DESKTOP III trial can prospectively assess the role of secondary CRS in patients with platinum-sensitive ovarian cancer. This important trial should answer several key questions regarding the management of recurrent EOC, including whether secondary CRS results in better outcomes than second-line chemotherapy.

## Abbreviations

CCR: Complete clinical response; OS: Overall survival; OR: Odds ratio; CI: Confidence interval; ROC: Receiver operating characteristic; EOC: Epithelial ovarian cancer; CRS: Cytoreductive surgery; ULN: Upper limit of normal; RECIST: Response Evaluation Criteria in Solid Tumors; WHO: World Health Organization.

## Competing interests

The authors declare that they have no competing interests.

## Authors’ contributions

XXC and FW participated in drafting the full manuscript and writing of this manuscript. FW and YFY partly participated in clinical study design, coordination and data analysis. XX and XHZ participated in creating figures. XX and YFY contributed by writing specific sections of this manuscript. XHZ and JHW provided advice and participated in revising the manuscript. XXC participated in substantial contribution to conception and revising it critically for important intellectual content. All the authors in this manuscript have read and approved the final version.

## Supplementary Material

Additional file 1: Figure S1 Patients who underwent secondary CRS had longer OS durations than those who did not undergo secondary CRS (A). Patients who underwent optimal secondary CRS had longer OS durations than those who underwent suboptimal surgery (B). Click here for file

Additional file 2: Figure S2 Patients who underwent secondary CRS had longer PFS durations than those who did not undergo secondary CRS (A). Patients who underwent optimal secondary CRS had longer PFS durations than those who underwent suboptimal surgery (B). Click here for file
